# Draft Genome Sequence of Cyclic Lipopeptide Producer *Pseudomonas* sp. Strain SWRI103, Isolated from Wheat Rhizosphere

**DOI:** 10.1128/MRA.00538-20

**Published:** 2020-07-02

**Authors:** Samira Zarvandi, Tahereh Bahrami, Brent Pauwels, Ahmad Asgharzadeh, Mehdi Hosseini-Mazinani, Farzaneh Salari, Léa Girard, René De Mot, Hassan Rokni-Zadeh

**Affiliations:** a Department of Medical Biotechnology, School of Medicine, Zanjan University of Medical Sciences, Zanjan, Iran; b Department of Microbial and Molecular Systems, Faculty of Bioscience Engineering, University of Leuven, Heverlee-Leuven, Belgium; c Agricultural Research, Education and Extension Organization (AREEO)-Soil and Water Research Institute, Karaj, Iran; d National Institute of Genetic Engineering and Biotechnology (NIGEB), Tehran, Iran; e Department of Mathematics and Computer Science, Amirkabir University of Technology, Tehran, Iran; f Zanjan Pharmaceutical Biotechnology Research Center, Zanjan University of Medical Sciences, Zanjan, Iran; University of Arizona

## Abstract

The draft genome sequence of wheat rhizosphere isolate *Pseudomonas* sp. strain SWRI103 is reported. This strain carries several gene clusters encoding nonribosomal peptide synthetases (NRPSs), including a system for cyclic lipopeptide (CLP) production, and genes for carotenoid biosynthesis.

## ANNOUNCEMENT

To identify siderophore-producing pseudomonads, a collection of around 200 fluorescent *Pseudomonas* strains was isolated from 52 rhizosphere samples of 21 wheat cultivars obtained from 10 different regions in Iran (1). Subsequently, their potential for *in vitro* bacterial antagonism was assessed (2). In addition to this phenotype-based strategy, a PCR-based approach was developed to detect candidate producers of lipopeptides (LPs) among these isolates (3). LPs are a diverse group of secondary metabolites synthesized by nonribosomal peptide synthetases (NRPSs), showing a wide range of biological activities, such as antimicrobial properties. Sequencing of amplicons obtained by targeting the lipoinitiation and tandem thioesterase domains of *Pseudomonas* NRPS genes identified *Pseudomonas* sp. strain SWRI103 as a candidate LP producer. This strain was isolated from the rhizosphere of wheat (variety Azadi) grown in the Shiraz region of Iran. Genomic sequencing will facilitate the identification of the LP biosynthetic cluster and characterization of its product. Default parameters were used for all software without exception.

Strain SWRI103, obtained from the Culture Collection for Soil Microorganisms (CCSM; Soil and Water Research Institute [SWRI], Iran), was cultured in one subculture in Trypticase soy broth (TSB) or Trypticase soy agar (TSA) medium (Laboratorios CONDA, Spain) at 30°C.

For genomic DNA isolation from the pure broth culture, the Qiagen Gentra Puregene Yeast/Bact kit was used. The shotgun library preparation was performed using a TruSeq Nano DNA library prep kit with a target insert size of 350 bp (Illumina, San Diego, CA). Paired-end sequencing (2 × 101-bp paired-end reads) was performed with an Illumina HiSeq 2000 system at Macrogen (Seoul, South Korea). A total of 4,729,067 paired-end reads were generated. FastQC version 0.11.5 was used to assess the quality of the reads, and all plots and reports passed the required threshold, displaying approved quality of sequencing. The *de novo* assembly was performed using Velvet version 1.2.10 with default parameters. A total of 245 contigs with an *N*_50_ value of 542,034 bp (about 130-fold coverage) were generated. The final assembled length comprises 6,038,401 bp with a G+C content of 60.8% and a longest contig size of 146,606 bp. Annotation of the assembled contigs using the NCBI Prokaryotic Genome Annotation Pipeline version 4.11 (4) identified 5,432 coding DNA sequences, 32 tRNA genes, and an *ssrA* transfer-messenger RNA (tmRNA) gene.

Analysis of biosynthetic gene clusters (BGCs) of secondary metabolites using antiSMASH 5.0 (5) revealed a BGC with three NRPS genes that show similarity to the cyclic lipopeptide (CLP) system of Pseudomonas fluorescens BW11P2 for production of bananamides (GenBank accession number KX437753), with 74.1% amino acid identity for the three concatenated biosynthetic enzymes (6). Another NRPS gene (*pvfC*) is located in a BGC syntenic to the Pseudomonas entomophila *pvfABCD* operon that encodes enzymes for the biosynthesis of pyrazine-*N*-oxides (7). These signal molecules are involved in the production of the pore-forming toxin monalysin by the insect pathogen *P. entomophila* (8) and of the phytotoxin mangotoxin by Pseudomonas syringae (9), as well as in biocontrol activity of Pseudomonas fluorescens (10). *Pseudomonas* sp. SWRI103 also encodes homologues of the *lpiBC* and *braBC* genes, suggesting the capacity to produce a cyclocarbamate type of antibiotic (11, 12). In addition, a complete carotenoid biosynthesis gene cluster is present (13, 14).

The draft genome sequence of *Pseudomonas* sp. SWRI103 reported here provides a valuable resource for studying its secondary metabolite production.

### Data availability.

This whole-genome shotgun project has been deposited at DDBJ/ENA/GenBank under the accession number JABBCM000000000. The version described in this paper is version JABBCM010000000. The raw sequencing data are available from the Sequence Read Archive (SRA) under the accession number PRJNA623691.
